# Phenotypic and functional characterization of natural killer cells in rheumatoid arthritis-regulation with interleukin-15

**DOI:** 10.1038/s41598-020-62654-z

**Published:** 2020-04-03

**Authors:** Syh-Jae Lin, Chien-Ya Hsu, Ming-Ling Kuo, Pei-Tzu Lee, Hsiu-Shan Hsiao, Ji-Yih Chen

**Affiliations:** 1 Division of Asthma, Allergy, and Rheumatology, Department of Pediatrics, Chang Gung Children’s Hospital, College of Medicine, Chang Gung University, Taoyuan, Taiwan; 2 Department of Medicine, Division of Allergy, Immunology and Rheumatology, Chang Gung Memorial Hospital, Chang Gung University College of Medicine, Taoyuan, Taiwan; 3 Department of Microbiology and Immunology, Graduate Institute of Biomedical Sciences, College of Medicine, Chang Gung University, Tao-Yuan, Taiwan; 4 Chang Gung Immunology Consortium, Chang Gung Memorial Hospital and Chang Gung University, Tao-Yuan, Taiwan

**Keywords:** Interleukins, Rheumatoid arthritis

## Abstract

Rheumatoid arthritis (RA) is an autoimmune disease characterized by synovial inflammation and joint destruction. Previous studies have shown that natural killer (NK) cells may play an important role in the pathogenesis of RA. Interleukin (IL)-15, a pro-inflammatory cytokine which induces proliferation and differentiation of NK cells, is overexpressed in RA. In this present study, we examine various NKRs and adhesion molecule expression on NK cells from RA patients and their response to IL-15 stimulation. We also sought to study cytokine-induced memory-like (CIML) NK cells in RA patients. We established that 1. RA patients had higher NK cell percentages in peripheral blood and their serum IL-15 levels were higher compared to healthy volunteers; 2. NK cells from RA patients showed lower NKp46 expression and an impaired CD69 response to IL-15; 3. NK cells from RA patients showed higher CD158b and CD158e expression but lower CD62L expression; 4. exogenous IL-15 up-regulated CD69, CD158b, CD158e but down-regulated NKp46 and CD62L expression in RA; 5. As to CIML NK cells, restimulation - induced NK cytotoxicity and IFN-γ production was impaired in RA patients, 6. Reduced NKp46, perforin, and granzyme B expression on NK cells was found in RA patients with bone deformity and erosion, 7. RA disease activity (DAS28) showed inverse correlation with the percentages of CD56^+^CD3^−^ NK cells, and NKp46 and perforin expression on NK cells, respectively. Taken together, our study demonstrated differential expression of various NK receptors in RA patients. NKp46, CD158e, and perforin expression on NK cells may serve as markers of RA severity.

## Introduction

Rheumatoid arthritis (RA) is a chronic autoimmune disease characterized by the inflammation of synovial membrane and destruction of joints^1,2^. NK cells, defined by expression of CD56 and lack of CD3, are traditionally regarded as the member of innate immunity providing anti-tumor and anti-viral defense^3,4^. NK cells may also play a pathogenic role in autoimmune diseases, as several reports have shown accumulation of NK cells in inflammatory joints of RA and other arthritic diseases^5,6^.

Interleukin (IL)-15, a member of γ-chain cytokine, is a pro-inflammatory cytokine essential in NK cell proliferation and differentiation. Higher IL-15 levels are detected in serum and synovium of RA patients^7,8^. Cytokine-induced memory-like (CIML) NK cells refers to NK cells,when pre-activated with IL-12, IL-15, and IL-18, may exhibit enhanced function when re-activated following a resting period cultured with low dose IL-15^9^. CIML NK cells have enhanced anti-leukemic and anti-tumor capacity over conventional NK cells^10,11^. However, the role of CIML NK cells in autoimmunity remains to be determined.

In this present study, we perform a phenotypic and functional characterization of NK cells in RA patients and their response to IL-15 stimulation. We also sought to examine CIML NK cells in RA patients compared with healthy volunteers. Finally, we determine whether various NK cell surface markers correlated with RA disease severity.

## Results

### Characteristics of RA patients and healthy volunteers

The clinical characteristics of RA patients and healthy volunteers are shown in Table 1. RA patients were predominantly female (M:F = 9: 23), and age ranged from 22 to 84 years. Patients had higher CD56^+^CD3^−^ NK cell percentages in the peripheral blood compared with healthy controls (14.8 ± 1.5% vs. 7.7 ± 0.8%, *p* = 0.001).

**Table 1:** Clinical and laboratory characteristics of patients with rheumatoid arthritis (RA) and healthy volunteers.

Characteristics	Healthy volunteers (N = 20)	RA (N = 32)
Sex (male/female)	5/15	7/25
Age, Median (Range)	31 (21–47)	54 (22–84)
Das28 (Median and Range)	NA	4.7 (1.39–8.46)
RF (Unit/ml)	NA	100 (5–1200)
ESR, Median (Range, mg/dl)	NA	37.5 (4–99)
HS-CRP, Median (Range, mg/dl)	NA	16.35 (1.42–105.76)
Disease duration, Median (Range, years)	NA	12 (0.5–35)
**Medication**		
Prednisolone (5–7.5 mg/day)	NA	53.1%
Disease modifying anti rheumatic drugs (DMARDs)	NA	100%
Anti-tumor necrosis factor alpha agents (anti-TNF-α)	NA	28.1%

### Percentages of CD56^+^CD3^−^ NK cells and serum IL-15 on RA

**Figure 1:**
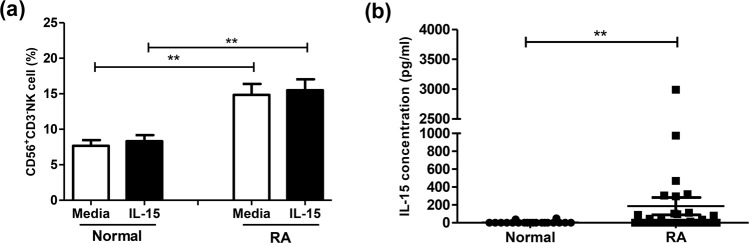
Comparison of (**a**) the percentages of peripheral blood CD56^+^CD3^−^ NK cells from RA patients (*n* = 32) and healthy volunteers (*n* = 20) under the influence of IL-15. MNCs were stimulated with or without IL-15 (10 ng/mL) for 18 hours and further stained by anti-CD3 and anti-CD56 antibodies and assessed by flow cytometry, and (**b**) serum IL-15 concentration of healthy volunteers (*n* = 20) and RA patients (*n* = 32). Data was presented as percent expression (%) ± SEM. ** p < 0.01, RA patients compare with healthy volunteers (Normal).

As shown in Fig. 1, RA patients had increased CD56^+^CD3^−^ NK cell percentages compared to healthy volunteers (14.8 ± 1.5% vs. 7.7 ± 0.8%, *p* = 0.001). Addition of IL-15 did not affect the percentages of CD56^+^CD3^−^ NK cells in both RA patients and healthy volunteers (RA 15.5 ± 1.5% vs. 14.8 ± 1.5%, *p* = 0.399; healthy volunteers 8.3 ± 0.9% vs. 7.7 ± 0.8%, *p* = 0.14). RA patients had higher serum IL-15 levels compared to normal control (186.2 ± 97.0 pg/ml vs. 4.4 ± 3.0 pg/ml, *p* = 0.009). Serum IL-15 level was higher in RA patients with DAS28 > 5.1 (308.6 ± 158.9 pg/ml) than those with DAS28 < 5.1 (7.2 ± 4.2 pg/ml) (*p* = 0.006). There was no significant correlation between CD3-CD56^+^ NK cells and IL-15 serum concentration in RA patients (Pearson correlation r = −0.08, *p* value = 0.461).

### Decreased NKp46, CD62L, but increased CD158b, CD158e expression on RA NK cells

**Figure 2:**
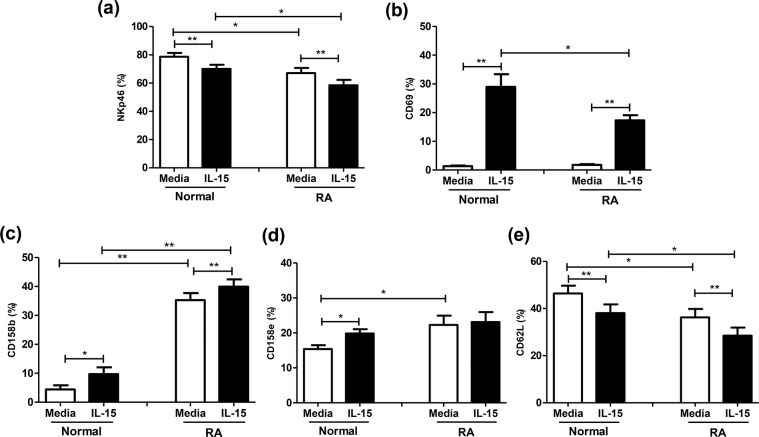
NKp46 (**a**), CD69 (**b**) CD158b (**c**), CD158e (**d**), and CD62L (**e**) expression of CD56^+^CD3^−^ NK cells from RA patients and healthy volunteers(Normal) with or without exogenous IL-15. MNCs were stimulated with or without IL-15 (10 ng/mL) for 18 hours, and further stained by anti-CD3 and anti-CD56 antibodies. CD56^+^CD3^−^ NK cells were gated and surface markers (NKp46, CD69, CD158b, CD158e, CD62L) expression were analyzed by flow cytometry. Data was presented as percent expression (%) ± SEM. ((**a**) healthy volunteers *n* = 17, RA *n* = 32 (**b**) healthy volunteers *n* = 20, RA *n* = 32 (**c**) healthy volunteers *n* = 7, RA *n* = 32 (**d**) healthy volunteers, *n* = 8, RA, *n* = 11 (**e**) healthy volunteers, *n* = 20, RA, *n* = 21). *means p < 0.05, **p < 0.01.

We next examined various activating and inhibitory NK receptor expression in RA patients, and the effect of IL-15 stimulation. NKp46 and CD69 are considered activating NK receptors. As shown in Fig. 2, NKp46 expression on NK cells was decreased in RA patients compared to healthy volunteers (67.1 ± 3.5% vs. 78.7 ± 2.6%, *p* = 0.029). IL-15 down-regulated NKp46 expression on NK cells from both RA patients (58.5 ± 3.8% vs. 67.1 ± 3.5%, *p* < 0.001) and healthy controls (70.1 ± 2.9% vs. 78.7 ± 2.6%, *p* = 0.001). CD69 expression on RA NK cells was comparable to healthy volunteers However, RA NK cells showed impaired response to exogenous IL-15 (17.3 ± 1.8%) compared to corresponding healthy volunteers (28.9 ± 4.5%, *p* = 0.036).

CD158b and CD158e are inhibitory NK receptors. As shown Fig. 2C, NK cells from RA patients expressed higher CD158b levels compared to healthy volunteers (35.3 ± 2.4% vs. 4.4 ± 1.5%, *p* < 0.001). Exogenous IL-15 up-regulated CD158b expression on both NK cells from RA patients (40.0 ± 2.5% vs. 35.3 ± 2.4%, *p* < 0.001) and healthy volunteers (9.7 ± 2.4% vs. 4.4 ± 1.5%, *p* = 0.018). The expression of CD158e on NK cells from RA patients was also higher than those from healthy volunteers (22.3 ± 2.7% vs. 16.3 ± 1.3%, *p* = 0.037). IL-15 enhanced the CD158e expression of healthy volunteers (19.8 ± 1.1% vs. 16.3 ± 1.3%, *p* = 0.028) but not in RA patients (RA 23.1 ± 2.9% vs. 22.3 ± 2.7%, *p* = 0.382)

CD62L is an adhesion molecule presented on NK cell that marks a mature NK subset with the ability to accumulate in inflamed tissues^12^. The CD62L expression on peripheral blood NK cells from RA patients was decreased compared to healthy volunteers (36.3 ± 3.5% vs. 46.4 ± 3.4%, *p* = 0.015). IL-15 down-regulated CD62L expression on NK cells from healthy volunteers (38.1 ± 3.7% vs. 46.4 ± 3.4%, *p* < 0.001) as well as RA patients (28.5 ± 3.4 vs. 36.3 ± 3.5%, *p* = 0.001).

### CIML NK cells from RA patients are deficient

We next determined whether high circulating IL-15 levels in RA patients would affect the generation of CIML NK cells. MNCs from RA patients and healthy volunteers were stimulated with IL-12 + IL-15 + IL-18 (pre-activated group) or IL-15 only (control group) for 16 hr. Cells were then washed and cultured with low-dose IL-15 (1 ng/ml) for 15days, after which cells were re-stimulated with IL-12 + IL-15 + IL-18 for 4 hours and harvested for assay.

**Figure 3:**
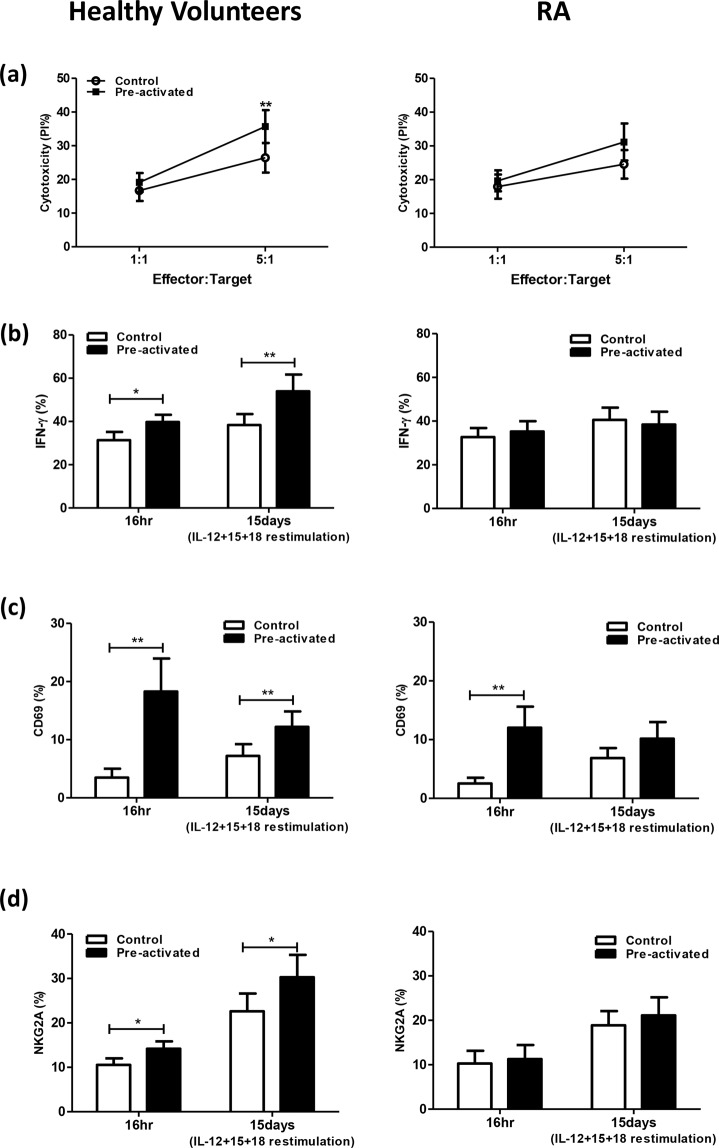
(**a**) K562 cytotoxicity, (**b**)IFN-γ secretion, (**c**) CD69 expression (**d**) NKG2A expression of cytokine-induced memory-like (CIML) NK cells from RA patients and healthy volunteers. Data was presented as percent expression (%) ± SEM. ((**a**) healthy volunteers *n* = 15, RA *n* = 12 (**b**) healthy volunteers *n* = 15, RA *n* = 11 (**c**) healthy volunteers *n* = 15, RA *n* = 12 (**d**) healthy volunteers *n* = 14, RA *n* = 10). * means p < 0.05, ** means p < 0.01.

Figure 3a shows the cytotoxicity against K562. Preactivated cells from healthy volunteers showed higher cytotoxicity than cells without pre-activation (36.9 ± 5.2 vs. 26.9 ± 4.7 p = 0.001 at E:T ratio of 5:1), suggesting of the presence of CIML NK cells. However, Preactivated cells from RA patients show comparable cytotoxicity compared to cells without pre-activation (31.2 ± 5.3% vs. 24.5 ± 4.2%, *p* = 0.071 at E:T ratio of 5:1).

Enhanced IFN-γ production of NK (CD3^−^/CD56^+^) cells were observed in pre-activated MNCs compared to controls (no pre-activation) from healthy volunteers (54.0 ± 7.7% vs. 38.4 ± 5.1%, *p* = 0.009). However, preactivated cells from RA patients failed to show increased NK IFN-γ expression after re-stimulation compared to controls (38.5 ± 5.8% vs. 40.5 ± 5.6%, *p* = 0.79) (Fig. 3b). Similarly, re-stimulation induced higher NK CD69 expression on preactivated MNCs from healthy volunteers compared to controls without preactivation (12.2 ± 2.6% vs. 7.2 ± 2.0%, *p* = 0.005). There was no difference of NK CD69 expression between preactivated groups and controls in RA patients. (10.2 ± 2.8% vs. 6.9 ± 1.7%, *p* = 0.168) (Fig. 3c).

Enhanced CD94/NKG2A expression of NK (CD3^−^/CD56^+^) cells were observed in pre-stimulated MNCs compared to controls (no pre-activation) from healthy volunteers (30.3 ± 5.0% vs. 22.6 ± 4.0%, *p* = 0.026). However, preactivated cells from RA patients failed to show increased NK NKG2A expression after re-stimulation compared to controls. (21.1 ± 4.1% vs.18.9 ± 3.2%, *p* = 0.241) (Fig. 3d).

We have also examined the IFN-γ response of the CD3^−^CD16^+^ CD56 ^low^, the more cytotoxic NK subsets in RA patients and controls. (Supplementary Fig. 1). The IFN-γ expression of CD3^−^CD16^+^CD56^low^ CIML NK cells showed similar trends to that observed with CD3^−^CD56^+^ CIML NK cells.

### Correlation of NK cell receptor expression and DAS28 disease activity

**Figure 4:**
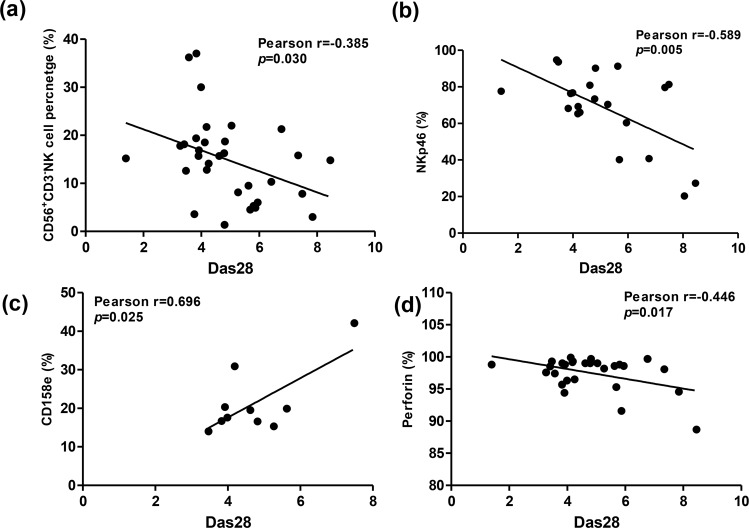
Correlation between (**a**) percentages of CD56^+^CD3^−^ NK cells and DAS28 score, *n* = 32; (**b**) NKp46 expression on NK cells and DAS28 score, *n* = 21; (**c**) CD158e expression on NK cells and DAS28 score, *n* = 10; (**d**) Perforin production of NK cells and DAS28 score, *n* = 28.

As shown in Fig. 4, the percentages of CD56^+^CD3^−^ NK cells showed inverse correlation with RA disease activity DAS28 (r = −0.385, *p* = 0.030). The DAS28 was also inversely correlated to NKp46 and perforin expression on RA NK cells, respectively (NKp46 r = −0.589, *p* = 0.005; Perforin r = −0.446, *p* = 0.017). However, the DAS28 showed positive correlation with CD158e expression on NK cells (r = 0.696, *p* = 0.025).

We next examined the NK receptor expression in RA patients with advanced disease including bone erosion and deformity. As shown in Table 2, NKp46 expression on NK cells from RA with deformity and erosion was lower than those from the controls. (30.0 ± 9.7% vs. 71.2 ± 4.1%, *p* = 0.003).

**Table 2:** Comparison of NKp46, CD69, CD158b, CD158e, CD62L, perforin (MFI), and Granzyme B (MFI) expression on CD56+CD3− NK cells from RA without deformity and erosion (controls) and RA with deformity and erosion.

	RA without bone erosion (Controls)	RA with bone erosion	*p* value
NKp46 (%)	71.2 ± 4.1	30.0 ± 9.7	0.003^**^
CD69 (%)	2.7 ± 0.6	0.7 ± 0.4	0.004^**^
CD158b (%)	32.7 ± 4.7	26.3 ± 5.1	0.438
CD158e (%)	22.7 ± 4.0	14.1 ± 3.5	0.372
CD62L (%)	46.0 ± 8.4	41.7 ± 7.3	0.874
Perforin (MFI)	11868 ± 2510	3518 ± 569	0.001^**^
Granzyme B (MFI)	6588 ± 1657	1265 ± 411	0.003^**^

^*^means p < 0.05, **means p < 0.01, controls compare with RA with bone deformity.

CD69 expression of NK cells was lower in RA patients with deformity and erosion (0.7 ± 0.4%vs. 2.7 ± 0.6%, *p* = 0.004). Perforin (MFI) expression on NK cells from RA with deformity and erosion was apparently decreased compared to controls. (3518 ± 569 vs. 11868 ± 2511, *p* = 0.001). Granzyme B (MFI) expression on NK cells from RA with deformity and erosion was decreased than controls (1265 ± 411 vs. 6588 ± 1657, *p* = 0.003).

## Discussion

In the present study, we sought to examine the expression of various NK cell receptors from RA patients and determined their response to exogenous IL-15. We found the percentages of NK cells are increased in PBMC from RA patients compared to controls. Higher serum IL-15 levels in RA patients which may contribute to the observed NK cell increase. Jin *et al*. who showed that the level of IL-15 and IL-17 were significantly elevated in synovial fluid of rats and involved in the perpetuation of RA synovitis^13^. Overexpression of IL-15 correlated with the induction of IL-17, a cytokine known to stimulate synoviocytes to release inflammatory factors such as IL-6, IL-8, GM-CSF and PGE_2_^14^.

NKp46, a natural cytotoxicity receptor, mediates NK cell-induced cell lysis and provide an innate defense against intracellular pathogens, NKp46 also serve as a key factor in the pathogenesis of type 1 diabetes mellitus by recognition of pancreatic β cells ligands^15–17^. We observed NK cells from RA patients expressed lower NKp46 than those from controls, and IL-15 decreased NKp46 expression of NK cells from RA patients, similar to that observed in controls. NKp46 expression level on RA NK cells was negatively correlated with RA disease activity.

CD69, a triggering C-type lectin receptor that plays a crucial role in inducing NK-mediated cytotoxicity, involved in NK cell proliferation and served as a signal transmitting receptor in NK cells^18,19^. We found there was no difference between expression of CD69 on NK cells from RA patients and controls. However, we found that expression of CD69 on RA NK cells showed impaired response to IL-15. Guo *et al*. also demonstrated that CD69 expression of NK cells could be up-regulated in response to IL-15-superagonist stimulation^20^.

CD158b and CD158e are Ig-like inhibitory receptors on the surface of NK cells^21,22^. CD158b recognizes the group HLA-C alleles (HLA-Cw1,3,7 and 8), presenting Ser-77 and Asn-80 26, and CD158e corresponds to HLA Bw4 allele. Both of them down regulate NK cytotoxicity via preventing cell lysis^23,24^. We found higher CD158b expression level on NK cells from RA patients compared to controls, in discrepancy with Pridgeon *et al*. who showed that CD158b expression on NK cells from controls and RA patients were almost identical^25^. IL-15 up-regulated CD158b on RA NK cells and control NK cells to a similar extent.

CD62L is an adhesion molecule that may play a crucial role in mediating the NK cell recruitment to inflamed synovium in RA. We showed that CD62L expression was lower on RA NK cells compared to controls, and exogenous IL-15 further down-regulated the CD62L expression. Yang *et al*. showed that the shedding of CD62L from surface of anti-tumor T cells and acquisition of lytic activity, suggests CD62L may play a key role in T cell effector functions and anti-tumor activity^26^.

CIML NK cells have been shown to have enhanced antitumor effect compared to conventional NK cells in adoptive immunotherapy^27^. However, the role of CIML NK cells in the pathogenesis of autoimmunity remains unclear. We are the first to study CIML NK cells in RA patients and found deficient cytotoxicity and IFN-γ production in CIML NK cells of RA patients. The CD69 and NKG2A expression of RA CIML NK cells was also decreased compared to healthy volunteers. Human CD94/NKG2A is an inhibitory receptor that recognizes HLA-E and is expressed by NK cells^28^. The clinical significance of this finding remains to be determined. IFN-γ is considered protective in RA as clinical symptoms of RA patients were relieved after administration of IFN-γ^29^. Our finding that deficient IFN-γ production of CIML NK cells may contribute in part to RA pathogenesis.

Granzyme B is an apoptosis inducer of chondrocytes with natural killer cell-like cytotoxicity in RA^30^. Perforin is a 70 kDa glycoprotein which is responsible for pore formation on the cell membrane of target cells induced by NK cells^31^. RA patients with bone erosion or deformity showed decreased granzyme B, perforin and NKp46 expression compared to those without bone erosion and deformity. We hypothesized that peripheral blood perforin and granzyme B expressing NK cells with great cytotoxic potential may migrate to inflamed joints in RA patients with bone erosion or deformity.

Taken together, we have characterized various NK receptor expression in RA patients and their response to IL-15, aberrant CIML NK cells in RA, and the correlation of NK receptor expression with RA disease severity, Further studies will be need to explore the pathogenic role of NK cells in RA patients.

## Methods

### Study subject

Study subjects include 32 RA patients and 20 healthy volunteers, the clinical characteristics of patients are summarized in Table 1. They were recruited from the Out-patient Clinic,Division of Rheumatology, Department of Internal Medicine at Chang Gung Memorial Hospital, Linkou, Taiwan. All the RA patients were diagnosed by the same physician according to the revised criteria of ACR/EULAR for RA^32^. Disease severity of patients were evaluated by the disease activity score, DAS28, according to the method of Prevoo *et al*.^33^, a multifaceted index composed of the number of swollen joints and number of tender joints out of 28 joints, erythrocyte sedimentation rate, and global assessment of health. Patients were separated into two groups according to whether bone deformity or erosions of fingers and toes on chest X-Ray diagnosed by radiologists or not. Peripheral blood samples were obtained from healthy volunteers with the approval of Chang Gung Medical Foundation Institutional Review Board (IRB: 201700445B0C501) and the informed consents had been obtained from all of the donors.

### Cell culture

Peripheral blood was collected in sterile tubes containing heparin (20 units/ml of blood) and was processed within 24 hours of collection. Mononuclear cells (MNCs) were then separated from heparinized blood using Ficoll-Hypaque density gradient centrifugation. MNCs (1 × 10^6^/ml) were incubated for 18 hours in complete RPMI-1640 (with 10% Fetal calf serum) in the present or absence of rhIL-15 (10 ng/ml, Preprotech, Rocky Hill, USA) for subsequent analysis.

### Flow cytometric analysis of NK cell markers

Cell staining was performed as previously described^34^. Cells were washed in cold PBS with 2% FCS and 0.1% sodium azide and following stained with fluorescein isothiocyanate (FITC)-, phycoerythrin (PE)-conjugated anti-human monoclonal antibodies which were including anti-CD3/CD16 + CD56 (APC/FITC), NKp46, CD69, CD62L, CD158b, and CD158e (PE) from Becton-Dickinson for flow cytometric analysis. The fluorescent staining was analyzed on a Canto II (BD Biosciences) flow cytometer. Electronic gates were set to enable analysis of the fluorescence of the viable cell population according to FSC/SSC histograms following anti-CD3/ CD16 + CD56 stimulation. The percentage of cells stained with each monoclonal antibody was determined by comparing each histogram with one from control cells stained with FITC- or PE- labeled isotype control monoclonal antibodies.

### CIML generation

For CIML generation, MNCs (3 × 10^6^/ml) were incubated with rhIL-12 (10 ng/ml, Preprotech, Rocky Hill, USA) plus rhIL-15 (1 ng/ml), IL-18 (50 ng/ml) for 16 hours as pre-activated group. MNCs were incubated with rhIL-15 (1 ng/ml) for 16hrs as control group. Control and pre-activated cells were wash 3 times, and cultured in RPMI-1640 with 10% human AB serum (Sigma-Aldrich) plus rhIL-15 (1 ng/ml) for 15 days. Every 2–3 days, half of the medium was discarded and replenished by fresh medium with IL-15 (1 ng/ml)^35^. Cultured cells were then harvested and stimulated with IL-12 (10 ng/ml), IL-18 (50 ng/ml) plus IL-15 (100 ng/ml) in the presence of GolgiStop protein transporter inhibitor containing monensin (BD Bioscience) for 4 hrs.

### Intracellular stain assay

Cells were stained with anti-CD56 (FITC) plus anti-CD3 mAbs (APC) (BD Bioscience) for 20 minutes. Cells were washed in PBS. Cells (1 × 10^6^) were resuspended in Cytofix/Cytoperm solution (BD Bioscience). After 20 min at 4 °C and one wash with Perm/wash buffer, then cells were stained with anti-human IFN-gamma (PE, BD Bioscience) for 20 minutes at room temperature. The samples were analyzed by flow cytometry, as previously described^36^.

### NK cytotoxicity assay

Flow cytometric NK cytotoxicity assays were performed as previously described^37^. Control and memory-like NK cells were harvested after a rest period of 15 days. Cells (Effectors) were restimulated with IL-12, IL-15 and IL-18, and co-cultured with K-562 cells (targets) at E:T ratio of 1:1 and 5:1 for 4 hours. After incubation, cells were stained with CD45-FITC (BD Bioscience) and propidium iodide (PI, 1 mg/ml, Sigma). Percentages of dead K562 cells are calculated from histograms showing fluorescent intensity of PI uptake of K562 cells after subtraction of background cell death.

We confirm that the above protocols and methods were performed in accordance with the relevant guidelines and regulations

### Statistical analysis

For statistical analysis, data of RA patients and healthy volunteers were compared between groups using the nonparametric Mann-Whitney U-test. Wilcoxon signed rank test was used for observing the difference of responses with or without exogenous IL-15. The data are presented by means ± standard error of mean (SEM). if the *p* value is less than 0.05, comparison of groups was considered significantly distinct.

## Supplementary information

Supplementary information.

Supplementary Dataset 1.

Supplementary Dataset 2.

Supplementary Dataset 3.

Supplementary Dataset 4.

Supplementary Dataset 5.
