# Supplementary material for: Phenotypic and functional characterization of natural killer cells in rheumatoid arthritis-regulation with interleukin-15
Source: Sci Rep. 2020 Apr 3;10:5858. doi: 10.1038/s41598-020-62654-z (PMC7125139; doi:10.1038/s41598-020-62654-z)
Supplement: Supplementary file 1 — Supplementary information. [file 41598_2020_62654_MOESM1_ESM.doc]

**Phenotypic and functional characterization of natural killer cells in rheumatoid arthritis-regulation with interleukin-15**

Syh-Jae Lin ^1*^, Chien-Ya Hsu^1^, Ming-Ling Kuo^1,3,4^, Pei-Tzu Lee ^1^, Hsiu-Shan Hsiao^1^, Ji-Yih Chen^2*^

^1^Division of Asthma, Allergy, and Rheumatology, Department of Pediatrics, Chang Gung Children’s Hospital, College of Medicine, Chang Gung University. Taoyuan, Taiwan.

^2^Department of Medicine, Division of Allergy, Immunology and Rheumatology, Chang Gung Memorial Hospital, Chang Gung University College of Medicine

^3^Department of Microbiology and Immunology, Graduate Institute of Biomedical Sciences, College of Medicine, Chang Gung University, Tao-Yuan, Taiwan

^4^Chang Gung Immunology Consortium, Chang Gung Memorial Hospital and Chang Gung University, Tao-Yuan, Taiwan

^+^Syh-Jae Lin and Hsu-Chien Ya contributed equally to this work and share the first authorship

Correspondence and address reprint to Dr. Syh-Jae Lin and Dr. Ji-Yih Chen

Dr. Syh-Jae Lin

Division of Allergy, Asthma, and Rheumatology

Department of Pediatrics

Chang Gung Children’s Hospital

5 Fu-Hsing Street, Kweishan, Taoyuan, Taiwan

FAX: 886-3-3288957

E-mail: [syhjaelin@gmail.com](mailto:syhjaelin@gmail.com)

Dr. Ji-Yih Chen

Department of Medicine, Division of Allergy, Immunology and Rheumatology, Chang Gung Memorial Hospital, Chang Gung University College of Medicine

5 Fu-Hsing Street, Kweishan, Taoyuan, Taiwan

FAX: 886-3-3288957

E-mail: jychen071688@gmail.com

**A.**

**B.**

Healthy Volunteers RA

**Supplementary Figure 1** IFN-r secretion of cytokine-induced memory-like (CIML) NK cells from RA patients and healthy volunteers. MNCs was incubated with rhIL-15 (1ng/ml) for 16 hours as control group, or incubated with rhIL-12 (10ng/ml) plus rhIL-15 (1ng/ml), IL-18 (50ng/ml) for 16 hours as pre-activated group. Control and preactivated cells were cultured with IL-15 (1ng/ml) for 15 days. After 15 days, cultured cells were harvested and stimulated with rhIL-12 (10ng/ml) plus rhIL-15 (100ng/ml), IL-18 (50ng/ml) for 4 hours. **A.** Representative profile of intracellular IFN-γ staining of CD3^-^CD16^+^CD56^low^ cells (Day15); **B.** Data was presented as percent IFN-γ secretion (%)± SEM. Healthy volunteers *n*=12, RA *n*=8.
